# Type III Kounis Syndrome Caused by Iodine Contrast Media After Improvement of Allergic Symptoms

**DOI:** 10.7759/cureus.55514

**Published:** 2024-03-04

**Authors:** Ryuichiro Okuda, Shu Utsumi, Hideki Tanaka, Tatsuo Takama, Yasuyuki Kakihana

**Affiliations:** 1 Emergency and Critical Care Center, Kagoshima City Hospital, Kagoshima, JPN; 2 Emergency and Intensive Care Medicine, Kagoshima University Graduate School of Medical and Dental Sciences, Kagoshima, JPN; 3 Emergency and Critical Care Medicine, Graduate School of Biomedical and Health Sciences, Hiroshima University, Hiroshima, JPN; 4 Cardiology, National Hospital Organization Kagoshima Medical Center, Kagoshima, JPN

**Keywords:** anaphylactic shock, contrast-induced anaphylaxis, allergic reaction, iodine contrast media, kounis syndrome, extremely late stent thrombosis, iodinated contrast, anaphylax, kounis case study

## Abstract

Kounis syndrome is an acute coronary syndrome (ACS) caused by an allergic reaction that almost always occurs immediately and simultaneously with allergic symptoms. We present a case of Kounis syndrome type III that developed after complete resolution of contrast-induced anaphylaxis in a 60-year-old man with a coronary stent placed in the proximal left anterior descending (LAD) artery branch for ischemic heart disease. Contrast-enhanced computed tomography revealed anaphylactic shock. Symptoms quickly improved with intramuscular adrenaline injection; however, chest pain appeared after approximately 30 min. ECG revealed ST-wave elevation in the precordial leads. Coronary angiography revealed acute stent thrombosis with total occlusion of the proximal LAD, and percutaneous coronary angioplasty was performed. We diagnosed Kounis syndrome based on the allergic symptoms and ACS. Because some cases of Kounis syndrome develop after anaphylactic symptoms have resolved, it is advisable to follow-up patients with allergic symptoms and pay attention to chest symptoms and ECG changes, especially when they have a history of noted or treated coronary artery disease.

## Introduction

Acute coronary syndrome (ACS) occurs in conjunction with an allergic reaction in Kounis syndrome [1]. This syndrome is categorized into three types [1,2]: Type I, which is caused by coronary artery spasms without a history of coronary artery disease; Type II, where there is a history of coronary artery disease and spasms lead to plaque erosion or rupture; and Type III, which is caused by thrombosis within a coronary artery stent. While cases of Kounis syndrome caused by contrast agents typically present with ACS immediately following an allergic reaction, we present a case in which Type III Kounis syndrome developed after the complete resolution of anaphylactic symptoms induced by a contrast agent [3,4].

## Case presentation

**Figure 1:**
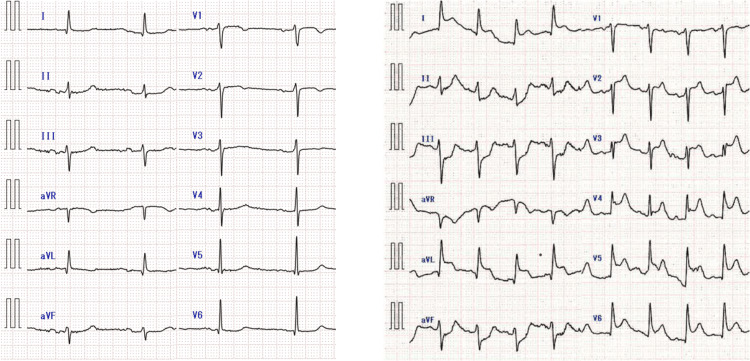
Electrocardiogram (ECG) changes A 12-lead ECG in the absence of chest symptoms after anaphylaxis shows no ST change, while a 12-lead ECG after the appearance of chest symptoms shows ST elevation at I, aVL, and V2-6.

**Figure 2:**
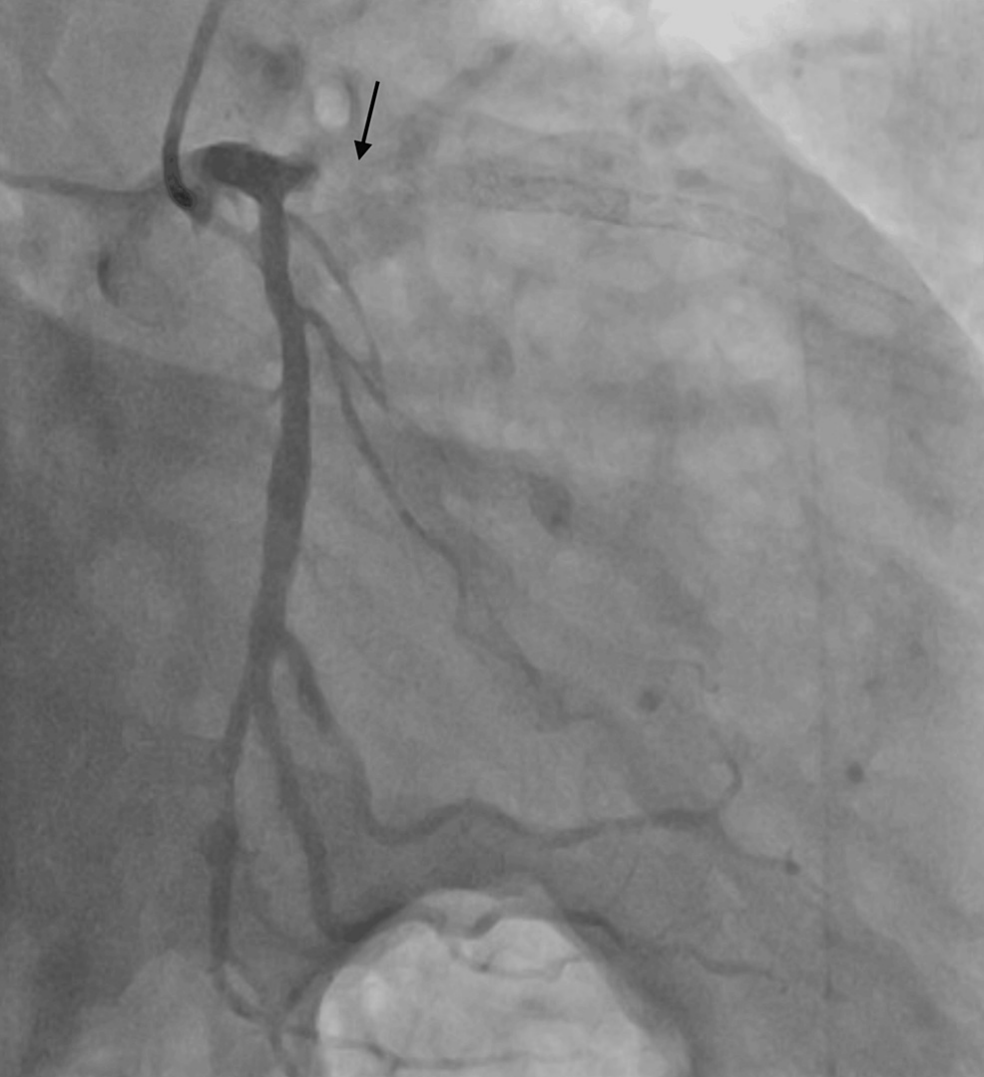
Coronary angiography Coronary angiography (caudal and right anterior oblique view) revealing left anterior descending (LAD) artery occlusion due to stent thrombosis.

A 60-year-old man with a coronary stent placed in the left anterior descending artery 17 years prior was diagnosed with an aortic aneurysm and underwent a contrast-enhanced computed tomography (CT) scan for evaluation in an outpatient setting. Immediately after the administration of the contrast agent iopamidol, the patient experienced a drop in blood pressure to approximately 70 mmHg and difficulty breathing. Anaphylactic shock was diagnosed, and the patient was treated with an intramuscular injection of adrenaline (0.5 mg) and a rapid infusion of saline. A few minutes later, his vital signs recovered and his symptoms completely disappeared. Although his respiratory and circulatory systems were stable, approximately 30 min after the disappearance of anaphylactic shock symptoms, he suddenly complained of chest discomfort. Electrocardiography showed ST changes (Figure 1), and echocardiography revealed reduced wall motion from the anterior septum to the apex. He was diagnosed with ischemic heart disease and treated with one sublingual tablet of nitropen (nitroglycerin) and a continuous administration of noradrenaline at 0.1 γ to treat his low blood pressure (79/49 mmHg). Emergency coronary catheterization revealed a complete occlusion of the proximal part of the stent in the left anterior descending artery (Figure 2). A percutaneous coronary intervention was performed, and the patient was admitted to the ICU. His condition improved, and he was transferred from the ICU to a general ward; he was then discharged on the 14th day.

## Discussion

Kounis syndrome is a condition in which allergic reactions occur simultaneously with ACS. Mediators released from mast cells during allergic reactions are believed to cause coronary vasoconstriction and acute myocardial infarction. First reported in 1991 by Kounis, it is also referred to as allergic angina [5]. Exposure to various substances such as drugs (antibiotics and nonsteroidal anti-inflammatories), contrast agents, bee stings, and certain foods can trigger mast cell degranulation and induce an allergic reaction, followed by the release of various inflammatory mediators. Kounis syndrome, which is associated with allergic reactions, is caused by the action of these mediators and cytokines, leading to coronary artery spasm, destabilization of coronary artery plaques, rupture, and thrombus formation, ultimately causing coronary blood flow disturbances [6].

Kounis syndrome is classified into three types. Type I occurs in patients with normal coronary arteries where the acute release of inflammatory mediators induces coronary artery spasms. In Type II, spasms in the coronary artery adjacent to an existing atherosclerotic lesion lead to plaque erosion or rupture. Type III is associated with coronary stent thrombosis in patients undergoing stenting. As the acute release of inflammatory mediators induces coronary artery spasms, Types I and II Kounis syndrome, which are primarily caused by coronary spasms, are likely to present with ACS almost simultaneously with allergic symptoms.

Kounis syndrome incidence is often underestimated because it is rare, meaning its actual incidence may be higher than that currently reported. We searched the literature on PubMed up to October 31, 2023, to investigate the relationship between the time from contrast agent administration to the onset of allergic symptoms and from the onset of allergic symptoms to the appearance of chest symptoms suggestive of ACS (Table 1).

**Table 1:** Summary of reports of contrast agent-related Kounis syndrome Delay①: From contrast administration to onset of allergic symptoms. Delay②: From the onset of anaphylaxis to the appearance of ACS-suspected symptoms.

Authors	Age/Sex	Delay①	Delay②	KS type	Cardiac arrest	Region
Wong et al., 2023 [3]	56/M	Immediately	Immediately	Ⅰ	no	Singapore
Prisco et al., 2020 [4]	53/F	Immediately	Immediately	Ⅰ	yes	USA
Xie et al., 2019 [6]	72/F	20 min	nr	nr	no	China
Xie et al., 2019 [6]	78/M	20 min	nr	Ⅱ	no	China
Choudhry et al., 2019 [7]	30/M	Immediately	Immediately, 16 hr	Ⅰ	no	USA
Maadarani et al., 2020 [8]	60/M	20 min	Immediately, 7 hr	Ⅲ	no	Kuwait
Singer et al., 2009 [9]	57/M	2 min	Immediately	nr	yes	USA
Dauvergne et al., 2009 [10]	79/M	Immediately	Immediately	Ⅱ	no	Chile
Kogias et al., 2010 [11]	48/M	10 min	Immediately	Ⅲ	no	Greece
Park et al., 2010 [12]	54/M	10 min	Immediately	Ⅱ	no	Korea
Kocabay et al., 2012 [13]	51/M	10 min	Immediately	Ⅱ	no	Turkey
Yanagawa et al., 2012 [14]	62/M	During infusion	Immediately	Ⅰ	yes	Japan
Zlojtro et al., 2013 [15]	46/F	10 min	Immediately	Ⅱ	no	Croatia
Xu et al., 2013 [16]	71/M	1 h	Immediately	Ⅰ	no	China
Benedetto et al., 2015 [17]	83/F	During infusion	Immediately	Ⅰ	no	Netherlands
Akita et al., 2016 [18]	70/M	Immediately	Immediately	Ⅰ	yes	Japan
Oh et al., 2016 [19]	74/M	Immediately	Immediately	Ⅰ	yes	Korea
Demoulin et al., 2017 [20]	81/M	Several min	Immediately	Ⅱ	yes	France
Tripolino et al., 2018 [21]	47/M	30 min	Immediately	Ⅲ	no	Italy
Bhaskaran et al., 2018 [22]	83/M	Immediately	Immediately	Ⅱ	no	Australia
Abusnina et al., 2019 [23]	53/F	Several min	Immediately	Ⅰ	no	USA
Dorniak et al., 2019 [24]	59/F	Within min	Within min	Ⅰ	no	Poland
Shibuya et al., 2019 [25]	60/M	Immediately	Immediately	Ⅱ	no	Japan
Tanaka et al., 2019 [26]	78/M	Immediately	Immediately	Ⅱ	no	Japan
Chien et al., 2019 [27]	81/F	15 min	Immediately	nr	yes	Taiwan
Biagioni et al., 2020 [28]	45/M	2 min	Immediately	Ⅰ	yes	Italy
Portero-Portaz et al., 2020 [29]	72/M	Immediately	Several min	Ⅲ	no	Spain
Elzeneini et al., 2020 [30]	76/M	Within min	Immediately	Ⅱ	yes	USA
Kangzheng et al., 2021 [31]	59/M	During infusion	nr	Ⅱ	no	China
Lee Chuy et al., 2022 [32]	59/M	Immediately	Immediately	Ⅰ	no	USA
Sagalov et al., 2023 [33]	51/M	Shortly after	Immediately	Ⅰ	no	USA
Singh et al., 2023 [34]	79/F	Immediately	nr	Ⅱ	no	USA
Our case	76/M	During infusion	37 min	Ⅲ	no	Japan

There have been 32 reports of contrast agent-related Kounis syndrome. Except for one case reported by Choudhry et al., chest symptoms occurred almost simultaneously with allergic symptoms or anaphylaxis. Choudhry et al. reported the case of a 35-year-old man who developed allergic symptoms (urticaria, chest tightness, and laryngeal spasm) after the administration of the contrast agent gadobenate dimeglumine [7]. Although the allergic symptoms temporarily improved, severe chest pain reappeared 16 hours later, and the troponin test was positive; however, echocardiography showed no local wall motion abnormalities, and angiography did not reveal any coronary artery stenosis. This was an atypical case of Type I Kounis syndrome.

Our case is a classic example of Type III Kounis syndrome, in which symptoms of anaphylaxis caused by the contrast agent initially completely disappeared; however, 30 min later, complete occlusion near the coronary artery stent was observed. According to previous reports, Kounis syndrome caused by contrast agents typically occurs almost simultaneously with allergic reactions immediately after administration of the contrast agent. Our case is the first reported instance in which typical Type III Kounis syndrome developed after the complete disappearance of allergic symptoms caused by a contrast agent.

Key inflammatory mediators released from mast cells such as histamine, leukotrienes, and prostaglandins initiate an amplifying cascade of blood coagulation, subsequently promoting thrombosis within the blood vessels [1]. In cases where a coronary artery stent is in place, the stent itself, drugs eluted from the stent, and administered antiplatelet drugs can chronically induce an inflammatory state in the stent intima, making it prone to stent thrombosis [35,36]. Coronary artery stent thrombosis, which progresses owing to inflammatory mediators, increases over time, and Kounis syndrome develops when the stent becomes completely occluded. This means that the onset of Type III Kounis syndrome may not occur simultaneously with allergic symptoms but can develop as a delayed reaction.

Maadarani et al. reported the case of a 60-year-old male with a drug-eluting stent who experienced severe chest pain, ST-segment elevation, and decreased wall motion 20 min after administration of a contrast agent [8]. Although thrombolytic therapy temporarily resolved the chest pain and ST elevation, a few hours later the patient presented with severe chest pain, decreased left ventricular wall motion, and extensive ST elevation due to acute stent thrombosis with complete occlusion of the proximal part of the left anterior descending artery. This suggests that thrombus formation driven by the released inflammatory mediators progressed and led to complete stent occlusion hours later. In cases of coronary artery stent implantation, even after the complete disappearance of chest symptoms, attention should be paid to the potential onset of Kounis syndrome.

## Conclusions

Kounis syndrome, which is caused by contrast agents, induces ACS almost simultaneously with an allergic reaction. In this case, we report the delayed onset of Type III Kounis syndrome after the allergic symptoms caused by the contrast agent had completely disappeared. Patients with allergic symptoms to contrast agents should be monitored for Kounis syndrome, especially in cases where a coronary stent is in place because delayed Type III Kounis syndrome can occur due to stent thrombosis even after the disappearance of allergic symptoms. Reports on Kounis syndrome induced by contrast agents are limited, and further investigation is necessary to better understand this condition.
